# A Proof of Concept of the Usefulness of a TDM-Guided Strategy for Optimizing Pharmacokinetic/Pharmacodynamic Target of Continuous Infusion Ampicillin-Based Regimens in a Case Series of Patients with Enterococcal Bloodstream Infections and/or Endocarditis

**DOI:** 10.3390/antibiotics11081037

**Published:** 2022-08-01

**Authors:** Milo Gatti, Sara Tedeschi, Filippo Trapani, Stefania Ramirez, Rita Mancini, Maddalena Giannella, Pierluigi Viale, Federico Pea

**Affiliations:** 1 Department of Medical and Surgical Sciences, Alma Mater Studiorum University of Bologna, 40138 Bologna, Italy; milo.gatti2@unibo.it (M.G.); sara.tedeschi5@unibo.it (S.T.); maddalena.giannella@unibo.it (M.G.); pierluigi.viale@unibo.it (P.V.); 2 Clinical Pharmacology Unit, Department for Integrated Infectious Risk Management, IRCCS Azienda Ospedaliero-Universitaria di Bologna, 40138 Bologna, Italy; 3 Infectious Diseases Unit, Department for Integrated Infectious Risk Management, IRCCS Azienda Ospedaliero-Universitaria di Bologna, 40138 Bologna, Italy; filippofabio.trapani@aosp.bo.it; 4 LUM Metropolitan Laboratory, AUSL Bologna, 40138 Bologna, Italy; stefania.ramirez@ausl.bologna.it (S.R.); rita.mancini@ausl.bologna.it (R.M.)

**Keywords:** ampicillin, continuous infusion, *Enterococcus faecalis*, endocarditis, bloodstream infections, TDM-guided dosing adjustment

## Abstract

(1) Objective: To describe the usefulness of a real-time therapeutic drug monitoring (TDM)-based strategy for optimizing pharmacokinetic/pharmacodynamic (PK/PD) target attainment of continuous infusion (CI) ampicillin-based regimens in a case series of patients affected by suspected or documented enterococcal bloodstream infections (BSIs) and/or infective endocarditis (IE). (2) Methods: Patients treated with CI ampicillin-based regimens for documented or suspected enterococcal BSI/IE who underwent real-time therapeutic drug monitoring (TDM)-based expert clinical pharmacological advice (ECPA) between June 2021 and May 2022 were retrospectively assessed. Ampicillin concentrations were determined at steady state, and the free fraction (*f*C_ss_) was calculated according to a plasma protein binding of 20%. The *f*C_ss_/MIC ratio was selected as the PD parameter for ampicillin efficacy and was defined as optimal for values between 4 and 8. The requirement for TDM-guided ampicillin dosing adjustments was assessed. (3) Results: Data for 12 patients with documented (n = 10) or suspected (n = 2) enterococcal infections (7 with BSIs and 5 with IE) were retrieved. The ampicillin PK/PD target was optimal over time in all of the 10 documented infections. None of the enterococcal BSIs persisted. Following the first real-time TDM-based ECPA, ampicillin dosage was decreased by >50% in 11 out of 12 patients (91.7%). (4) Conclusions: CI may be helpful in attaining aggressive ampicillin PK/PD targets in patients affected by enterococcal BSIs and/or IE. Administration of CI ampicillin after loading coupled with real-time TDM-based ECPA could be a valuable strategy for managing enterococcal infections.

## 1. Introduction

In the last three decades, severe enterococcal infections, especially infective endocarditis (IE), have been associated with substantially unchanged mortality rates ranging from 20% to 40% [1]. *Enterococcus faecalis* has higher virulence and a greater ability to produce biofilm compared to *Enterococcus faecium*, and these characteristics make treatment of *Enterococcus faecalis*-related infections challenging, regardless of a less frequent occurrence of multi-drug resistance [2]. 

Unfortunately, beta-lactam monotherapy lacks bactericidal activity against *Enterococcus faecalis*, so that implementing antimicrobial combination regimens is mandatory when dealing with *Enterococcus faecalis*-related deep-seated infections [1,3]. Historically, a combination of ampicillin with an aminoglycoside was considered the best therapeutic strategy [1]. However, the worryingly increasing rate of high-level aminoglycoside resistance (HLAR) in *Enterococcus faecalis* isolates over the last decade has led to the need for a paradigm shift in the treatment of these infections. Nowadays, dual beta-lactam therapy based on ampicillin plus ceftriaxone has emerged as an effective and safer alternative strategy, when dealing not only with HLAR *Enterococcus faecalis*-related infections but also with non-HLAR *Enterococcus faecalis*-related ones [4,5]. 

Ampicillin doses as high as 2g q4h by intermittent infusion are currently recommended for the management of *Enterococcus faecalis*-related IE or bloodstream infections (BSIs) [3]. Like other beta-lactams, ampicillin has a short elimination half-life (approximately 1 h), so that its time-dependent pharmacodynamic activity (associated with the percentage of time that the unbound concentration is maintained above the minimum inhibitory concentration (MIC) of the targeted pathogen during the dosing interval (%*f*T > MIC)) can be maximized by continuous infusion (CI) administration [6]. This strategy may allow the achievement of very aggressive pharmacokinetic/pharmacodynamic (PK/PD) targets using lower daily doses. At the same time, this approach may minimize fluctuations of serum levels and may avoid the high peaks associated with intermittent infusion that may cause neurotoxicity [7,8]. To date, clinical data assessing the role of CI ampicillin in the treatment of *Enterococcus faecalis* infections have been quite limited [9,10,11]. 

The aim of our study was to describe the usefulness of a real-time therapeutic drug monitoring (TDM)-based strategy for optimizing the PK/PD target attainment of CI ampicillin in a case series of patients affected by suspected or documented enterococcal BSIs and/or IE.

## 2. Results

Overall, during the study period, 12 patients had documented or suspected *Enterococcus faecalis* IE or BSIs treated with CI ampicillin-based regimens and underwent at least one instance of TDM-based expert clinical pharmacological advice (ECPA) with ampicillin concentration assessed in a steady state (C_ss_; Table 1).

The mean (± standard deviation (SD)) age was 69.3 ± 8.1 years, with a male preponderance (60%), and 2 out of the 12 patients (16.7%) required ICU admission. Mean (±SD) creatinine clearance (CLCr) at baseline was 44.0 ± 31.2 mL/min/1.73m^2^, and overall CLCr was <50 mL/min/1.73m^2^ in 6 out of the 12 patients (50.0%). Furthermore, continuous renal replacement therapy was applied in 1 case.

There were 7 patients with BSIs and 5 with IE. Of these, 4 BSIs were primary, whereas 2 were secondary to complicated urinary tract infections (cUTIs) and another was catheter-related. All BSIs were microbiologically documented and were caused by *Enterococcus faecalis* in 6 cases and by *Enterococcus casseliflavus* in the remaining case. IE was microbiologically documented in 3 out of 5 cases, and all were caused by *Enterococcus faecalis*. The other 2 cases were classified as possible IE based on modified Duke criteria [12]. Blood cultures were negative and were treated with ampicillin-based regimens according to a previous recent history of *Enterococcus faecalis*-related BSI. Overall, all the clinical isolates were fully susceptible to ampicillin, and the minimum inhibitory concentrations (MICs) ranged from 1 to 4 mg/L.

After a loading ampicillin dose of 2 g infused over 1 h, a CI maintenance dose (MD) was started, with 3g q6h over 6 h in 6 patients, 2 g q6h over 6 h in 3 patients and 1.5 g q6h over 6h in 3 patients. The median (interquartile range (IQR)) duration of treatment was 13.5 days (10–21.25 days). CI ampicillin-based regimens were used in monotherapy in 2 cases and combined with 2g q12h ceftriaxone in 9 cases or with 700 mg q48h daptomycin in 1 case. The median (IQR) average ampicillin free steady-state concentration (*f*Css) was 65.6 mg/L (30.0–87.5 mg/L). All of the 10 patients with documented enterococcal infections achieved optimal ampicillin PK/PD targets, with a median (IQR) average *f*Css/MIC of 32.6 (18.6–69.8).

**Figure 1:**
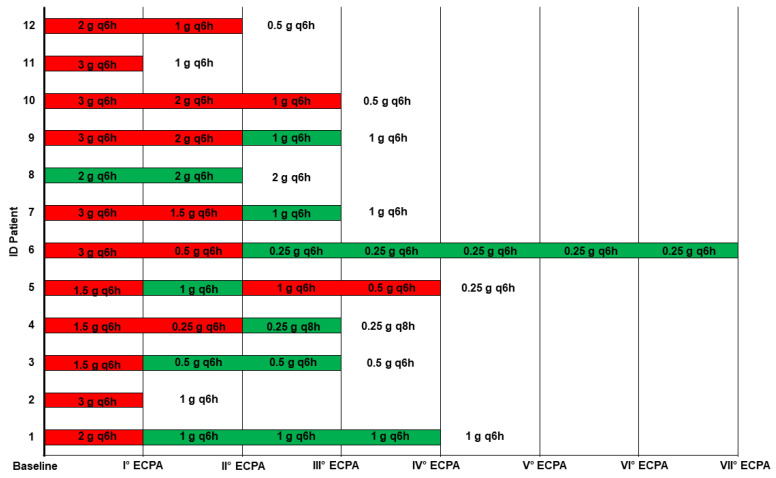
Recommended ampicillin dosing adjustments for each instance of TDM-guided expert clinical pharmacological advice in patients with documented or suspected enterococcal bloodstream infections and/or infective endocarditis. Green box: no recommended ampicillin dosing adjustment; red box: recommended ampicillin dosing reduction; ECPA: expert clinical pharmacological advice.

Overall, a total of 36 real-time instances of TDM-based ampicillin ECPA were provided, with a median (IQR) of 3 (2–3.25) per patient. At the first ECPA, an ampicillin dosing reduction > 50% was recommended in 11 out of 12 patients (91.7%) (Figure 1). 

Overall, ampicillin dosage decreases were recommended in 20 out of 36 ECPA cases (55.6%), whereas dosage increase was never needed. The patients’ individual courses of the ampicillin *f*C_ss_/MIC ratio and of the administered dosing regimens are summarized in Table 2.

None of the enterococcal BSIs persisted in patients with documented infections. The overall 90-day mortality rate was 25.0% (3/12). Specifically, mortality occurred in three patients with severe underlying diseases. Two of these were patients with IE who were not eligible for cardiac surgery due to considerable underlying comorbidities and severe cardiac failure, and the other was a critically ill patient with severe underlying COVID-19 pneumonia complicated by enterococcal superinfection. No ampicillin-related adverse events, including neurotoxicity, emerged during treatment.

## 3. Discussion

To the best of our knowledge, this is the first study to describe the usefulness of a real-time therapeutic drug monitoring (TDM)-based strategy for optimizing PK/PD target attainment of CI ampicillin in a real-world scenario of patients affected by enterococcal BSIs and/or IE. 

Administration of traditional beta-lactams by CI, by achieving more aggressive PK/PD targets, has been shown to grant remarkable advantages over intermittent infusion in terms of better microbiological outcomes and/or clinical outcomes in septic patients [7,13,14]. With regard to ampicillin, an experimental rat model of enterococcal IE showed that CI ampicillin significantly increased the survival rate of animals and the sterilization of both blood cultures and cardiac vegetations, compared to intermittent infusion [15]. However, clinical data supporting the use of CI ampicillin for enterococcal infections are limited to only few cases of patients affected by chronic infections who were included in outpatient parenteral antimicrobial therapy (OPAT) programs [9,10,11,16].

Our findings showed firstly that implementing a real-time TDM-guided ECPA program of CI ampicillin may be helpful in attaining very aggressive PK/PD targets over time. To date, the only case series that assessed the PK/PD target attainment of CI ampicillin included three OPAT patients, none of whom had enterococcal IE [11]. 

Our approach allowed prompt optimization of ampicillin treatment in a very challenging clinical scenario, i.e., patients with enterococcal infections and various degrees of renal dysfunction, which is a quite common occurrence among elderly patients with multiple comorbidities [1,17]. 

In this setting, our usual approach is to start with a loading dose of 2 g over 1 h, followed by an MD of 2–3 g q6h over 6h CI ampicillin in all of the patients, irrespective of the degree of renal function. This is thought to ensure prompt achievement of very aggressive PK/PD targets and to deal with the eventuality of a rapid recovery of renal function in patients with transient acute kidney injury, which might lead to underexposure in the first 24–48 h [18]. In this scenario, real-time TDM-guided ECPA, by allowing prompt dosing adjustments within 48 h, may be helpful in preventing prolonged ampicillin overexposure, which may lead to adverse events, namely neurotoxicity [19]. Although ampicillin is a fairly well-tolerated beta-lactam compared to other agents in the class [20], it should not be overlooked that ampicillin-related neurotoxicity risk may be increased in elderly patients with renal dysfunction, which is a particular feature of patients with severe enterococcal infections [17]. 

In this regard, it is worth mentioning that the CI ampicillin dosages needed for maintaining the optimal PK/PD target against enterococcal BSI and IE in our patients were quite low, as shown by the >50% dosage reductions recommended in the vast majority of the instances of TDM-guided ECPA. From the perspective of the antimicrobial puzzle [21], these findings could be explained by various aspects: the high susceptibility of the enterococcal clinical isolates with low MIC values of ampicillin, usually 1 mg/L; the added value of CI administration in allowing higher targets under the same daily dose compared to intermittent infusion; and the decreased clearance of ampicillin in patients with mild-to-moderate renal dysfunction, who were the most frequent types of cases in our series. In addition, this suggests that the ampicillin doses to be administered by CI for optimal treatment of elderly patients with enterococcal infections could be lower than those actually recommended for intermittent infusion. Obviously, population pharmacokinetic analysis and Monte Carlo simulations would allow us to clarify this issue.

Notably, persistence or recurrence are the most worrisome clinical features in enterococcal bloodstream infections. Indeed, persistence recently emerged as an independent predictor of 30-day mortality in patients affected by enterococcal BSI [22]. In this scenario, administering ampicillin by CI and implementing a real-time TDM-guided strategy may represent a valuable approach for maximizing the achievement of optimal PK/PD targets and consequently minimizing the risk of occurrence of persistent enterococcal BSI. Indeed, preclinical and clinical evidence showed that a C_ss_/MIC ratio, or even a trough concentration/MIC ratio, ≥4 may be associated with suppression of the emergence of resistance to beta-lactams [13,23]. 

Although our study simply represents a proof of concept of the usefulness of a real-time TDM-based strategy for optimizing the ampicillin PK/PD target, it should not be overlooked that no BSIs persisted in the patients with documented enterococcal infections. Clearly, this issue should be investigated in larger clinical studies.

Nowadays, the implementation of dedicated bundles for the management of Gram-positive BSIs has become the best therapeutic approach, showing a remarkable impact in terms of survival for both methicillin-resistant *Staphylococcus aureus* and enterococcal-related infections [24,25]. In this scenario, CI administration of ampicillin coupled with prompt maximization of PK/PD target attainment could potentially represent a significant additional step in the enterococcal management bundle, along with other already consolidated approaches such as infectious disease consultation, echocardiography, follow-up blood cultures, and early targeted antibiotic treatment [24]. 

We recognize that our study has some limitations. The retrospective monocentric study design and the small sample size should be acknowledged. However, we considered that the timeframe of one year was sufficient to represent a proof of concept of the valuable role that the program of real-time TDM-guided CI ampicillin-based regimens (established in our hospital in June 2021) may play in dealing with severe enterococcal infections. Total ampicillin concentrations were measured, but the free moieties were only estimated. The PK/PD target attainment of concomitant antibiotics, namely ceftriaxone or daptomycin, were not assessed. Conversely, as a point of strength, this is the first real-life study that has analyzed, by means of a real-time TDM-based ECPA, the PK/PD target attainment of CI ampicillin in patients affected by enterococcal BSIs and/or IE. 

## 4. Materials and Methods

Patients who received CI ampicillin for the management of documented or suspected enterococcal IE or BSIs at the IRCCS Azienda Ospedaliero–Universitaria of Bologna and who underwent TDM of ampicillin between June 2021 and May 2022 were retrospectively analyzed. Demographic and clinical/laboratory data were extracted for each patient. The type/site of infection, ampicillin dosage, treatment duration, co-treatment with other antibiotics, MIC of ampicillin, and TDM-based ECPA dosing adjustments were also collected. 

Documented enterococcal BSI was defined as the isolation of an *Enterococcus* spp. from blood cultures, whereas documented or suspected IE was defined according to modified Duke criteria [12]. Ampicillin monotherapy or combination therapy was prescribed at the discretion of the infectious disease consultant, according to the current clinical practice guidelines implemented at the IRCCS Azienda Ospedaliero-Universitaria of Bologna. Treatment was always started with a loading dose of 2 g over 1 h, followed by an MD administered by CI. For this purpose, aqueous solutions were reconstituted every 6–8 h and infused over 6–8 h due to stability concerns [26]. Initial MD regimens were 2–3 g q6h over 6h and were subsequently optimized by means of adaptive TDM-based ECPA. 

Blood samples for assessing ampicillin C_ss_ were first collected within 72 h from starting treatment after achieving steady-state conditions. Each patient received ECPA based on one single blood sample collected at steady state. Subsequent reassessments were performed at the discretion of the attending physician. Total serum ampicillin concentrations were measured by means of liquid chromatography–tandem mass spectrometry (LC–MS/MS), using a Shimadzu liquid chromatography system (Nexera X2 30AD) coupled with a mass spectrometer (Ab Sciex API 5500 Qtrap). The analytical method was developed by bioanalytical experts located at the Unique Metropolitan Laboratory of Bologna. Briefly, the diagnostics kits of calibration standards and quality control samples provided by Chromsystems for measuring ampicillin and other beta-lactams in serum/plasma samples by means of HPLC systems (3 PLUS1 Multilevel Plasma Calibrator Set, Catalog number 61028 and Plasma Control level I-II, Catalog number 0183-0184, Chromsystems Instruments & Chemicals GmbH, Munich, Germany) were used for this purpose. Internal validation was performed by testing the selectivity, accuracy, and potential carryover of ampicillin detection. The method’s reliability in detecting and quantifying ampicillin concentrations properly was verified by testing the performance against the specific Chromsystems plasma calibration standard (Catalog number 61003) present in each lot. For reliability, the relative standard deviation should be <15%.

Gradient separation chromatography was carried out using a Mass Tox TDM Master Column series A from Chromsystems (Catalog Number 92110), with mobile phase A consisting of 0.1% aqueous formic acid (VWR, LCC International, and Merck, KGaA Darmstadt, Germany, for water MS grade and formic acid, respectively), and mobile phase B consisting of 0.1 % formic acid in acetonitrile. The total run time was 5 min. The percentage of solvent B started at 2% (0.5 mL/min total flow), and in the first 3 min the gradient (A/B ratio) slowly changed from 98/2 (*v*/*v*) to 36/64 (*v*/*v*). After reaching 20/80 (*v*/*v*), it was kept stable for less than 30 s, before being finally returned to baseline conditions (98/2 (*v*/*v*)). The column was maintained at a temperature of 40 °C. Mass spectrometric acquisition for ampicillin identification was performed using an electrospray ionization probe operating in positive mode. A double transition was used (350.1–106.1 as the quantifier transition and 350.1–160.1). The optimized declustering potential was 66 V, and the collision energies for each transition were 25 and 35 V, respectively. The mass spectrum parameters were set at 30 arbitrary units for the curtain gas, medium mode for the collision gas, 5500 V for the ion spray voltage, 40 arbitrary units for both gas source 1 and gas source 2, and 550 °C for the source temperature. 

The method showed a linear regression (R ≥ 0.995) in the range of concentrations of the Chromsystems calibration kit (range from 3.26 to 57.8 mg/L for lot 2321), and the calibration points tested during the analysis showed an accuracy between 85 and 118%, as for the Chromsystem quality controls, which were processed twice during each analysis. Whenever samples had ampicillin concentrations above the upper limit of quantification (ULOQ), they were diluted with blank serum and subsequently reanalyzed. Sample treatment was performed by means of a serum protein precipitation procedure. Briefly, 20 µL of the patient’s serum was added to 180 µL of acetonitrile containing linezolid-d3 (Alsachim Shimadzu Corporation, Graffenstaden, France) as an internal standard at a concentration of 1000 ppb. After vortexing and centrifugation, 20 µL of the supernatant was diluted in 200 µL of mobile phase A, and 2 µL was injected for LC-MS/MS analysis. The real-time TDM-based ECPA was provided to clinicians via the intranet system within 6h of blood sample delivery to the lab.

The free fraction (*f*) of the ampicillin concentration was calculated by considering a 20% plasma protein binding, as reported in the literature [27]. The *f*C_ss_/MIC ratio was selected as the PK/PD parameter for best describing the time-dependent efficacy of ampicillin and was defined as optimal in the range from 4 to 8, quasi-optimal if between 1 and 4, suboptimal if <1, and supra-optimal if >10. Dosing reduction was performed whenever *f*C_ss_/MIC was supra-optimal, to minimize the risk of neurotoxicity [8]. In accordance with its lower risk of neurotoxicity compared to other beta-lactams [20], no dosing adjustment was recommended when *f*Css/MIC was between 8 and 10. These thresholds were based on the findings of in vitro/experimental animal models and of clinical studies showing that C_ss_/MIC ratios and/or trough concentration/MIC ratios ≥ 4 (i.e., aggressive PK/PD targets) may be associated with microbiological eradication and suppression of the emergence of resistance to beta-lactams, as opposed to conservative PK/PD targets (i.e., 40–70% *f*T_>MIC_), resulting from preclinical studies and commonly implemented in clinical trials [13,23,28]. In the case of empirical treatment, the EUCAST clinical breakpoint of ampicillin for *Enterococci* was selected as the reference MIC value [28]. Persistent enterococcal BSI was defined as the further isolation of the same species of *Enterococcus* spp. from blood cultures after at least 72 h of appropriate antibiotic therapy, as previously reported [22]. 

Descriptive statistics were used. Continuous data were presented as mean ± SD or median IQR, whereas categorical variables were expressed as a count and percentage. Statistical analysis was performed by means of MedCalc for Windows (MedCalc statistical software, version 19.6.1, MedCalc Software Ltd, Ostend, Belgium). The study was approved by the Ethics Committee of the IRCCS Azienda Ospedaliero-Universitaria of Bologna (no. 442/2021/Oss/AOUBo, approved on 28 June 2021).

## 5. Conclusions

In conclusion, our findings suggest that administering ampicillin by CI and adopting a strategy of real-time TDM-guided ECPA for prompt dosing adaptation may be very helpful in attaining very aggressive PK/PD targets and in optimizing treatment in patients affected by enterococcal BSIs and/or IE. Large prospective clinical studies are warranted for investigating the relationship between the achievement of optimal ampicillin PK/PD targets and clinical outcomes.

## Figures and Tables

**Table 1:** Demographic and clinical features of patients with documented or suspected enterococcal infections treated with continuous infusion ampicillin.

ID Cases	Age/Sex	Ward	Isolates	Ampicillin MIC (mg/L)	Initial Ampicillin Dosage	Average CLCr (mL/min/1.73m^2^)	Average *f*C_ss_ (mg/L)	Average *f*C_ss_/MIC	Combination Therapy	Dosing Adjustment	Treatment Duration (Days)	Persistent BSI	90-Day Mortality
**Endocarditis**													
#1	81/M	Internal medicine	*E. faecalis*	2	2 g LD 2 g q6h CI	53	30.1	15.6	Ceftriaxone 2 g q12h	Reduction	22	No	No
#2	64/M	Cardiosurgery	No isolate	NA	2 g LD 3 g q6h CI	11	160.8	NA	Daptomycin 700 mg q48h	Reduction	33	NA	No
#3	77/M	Infectious Disease Unit	*E. faecalis*	1	2 g LD 1.5 g q6h CI	31	29.84	29.84	Ceftriaxone 2 g q12h	Reduction	13	No	No
#4	67/M	Internal medicine	No isolate	NA	2 g LD 1.5 g q6h CI	13	107	NA	Ceftriaxone 2 g q12h	Reduction	10	NA	Yes
#5	62/F	Cardiosurgery	*E. faecalis*	1	2 g LD 1.5 g q6h CI	18	67.3	67.3	Ceftriaxone 2 g q12h	Reduction	29	No	Yes
**Bloodstream infections**													
#6	68/M	Cardiac ICU	*E. casseliflavus*	1	2 g LD 3 g q6h CI	47	70.6	70.6	None	Reduction	21	No	No
#7	72/M	Internal medicine	*E. faecalis*	1	2 g LD 3 g q6h CI	59	21	21	Ceftriaxone 2 g q12h	Reduction	10	No	No
#8	70/M	Urology	*E. faecalis*	1	2 g LD 2 g q6h CI	61	17.8	17.8	Ceftriaxone 2 g q12h	Confirmation	10	No	No
#9	56/F	Cardiosurgery	*E. faecalis*	1	2 g LD 3 g q6h CI	75	35.4	35.4	Ceftriaxone 2 g q12h	Reduction	14	No	No
#10	61/F	COVID ICU	*E. faecalis*	4	2 g LD 3 g q6h CI	CVVHDF	64	16	None	Reduction	10	No	Yes
#11	83/M	Internal medicine	*E. faecalis*	1	2 g LD 3 g q6h CI	33	148	148	Ceftriaxone 2 g q12h	Reduction	7	No	No
#12	71/F	Internal medicine	*E. faecalis*	1	2 g LD 2 g q6h CI	114	81	81	Ceftriaxone 2 g q12h	Reduction	14	No	No

BSI: bloodstream infection; CI: continuous infusion; CLCr: creatinine clearance; CVVHDF: continuous veno-venous hemodiafiltration; *f*C_ss_: free fraction steady-state concentration; ICU: intensive care unit; LD: loading dose; MIC: minimum inhibitory concentration; MRSE; methicillin-resistant *Staphylococcus epidermidis*; NA: not available.

**Table 2:** Patients’ individual courses of the free ampicillin steady-state concentrations/MIC ratios and of the administered dosing regimens.

ID Case	Day	Ampicillin	
		Dosage	*f*C_ss_/MIC
#1	3	2 g q6h	21.2
	7	1 g q6h	11.8
	11	1 g q6h	9.1
	13	1 g q6h	11.4
#2	3	3 g q6h	40.2
#3	3	1.5 g q6h	48.9
	6	0.5 g q6h	24.6
	8	0.5 g q6h	15.8
#4	3	1.5 g q6h	39.8
	6	0.25 g q6h	30.4
	8	0.25 g q8h	10.0
#5	3	1.5 g q6h	39.2
	8	1 g q6h	45.0
	11	1 g q6h	57.6
	14	0.5 g q6h	127.2
#6	3	3 g q6h	292.0
	6	0.5 g q6h	93.6
	10	0.25 g q6h	9.3
	13	0.25 g q6h	9.1
	17	0.25 g q6h	7.9
	19	0.25 g q6h	10.3
	21	0.25 g q6h	9.7
#7	2	3 g q6h	29.8
	4	1.5 g q6h	20.4
	7	1 g q6h	12.8
#8	3	2 g q6h	21.1
	6	2 g q6h	14.6
#9	2	3 g q6h	43.6
	4	2 g q6h	46.7
	7	1 g q6h	15.7
#10	3	3 g q6h	18.3
	6	2 g q6h	16.0
	8	1 g q6h	13.7
#11	3	3 g q6h	148.0
#12	3	2 g q6h	96.0
	6	1 g q6h	66.0

*f*C_ss_: free fraction steady-state concentration; MIC: minimum inhibitory concentration.

## Data Availability

Not applicable.

## References

[1] Beganovic M., Luther M.K., Rice L.B., Arias C.A., Rybak M.J., Laplante K.L. (2018). A Review of Combination Antimicrobial Therapy for Enterococcus faecalis Bloodstream Infections and Infective Endocarditis. Clin. Infect. Dis..

[2] Duprè I., Zanetti S., Schito A.M., Fadda G., Sechi L.A. (2003). Incidence of virulence determinants in clinical Enterococcus faecium and Enterococcus faecalis isolates collected in Sardinia (Italy). J. Med Microbiol..

[3] Habib G., Lancellotti P., Antunes M.J., Bongiorni M.G., Casalta J.-P., Del Zotti F., Dulgheru R., El Khoury G., Erba P.A., Iung B. (2015). 2015 ESC Guidelines for the management of infective endocarditis: The Task Force for the Management of Infective Endocarditis of the European Society of Cardiology (ESC). Endorsed by: European Association for Cardio-Thoracic Surgery (EACTS), the European Association of Nuclear Medicine (EANM). Eur. Heart J..

[4] Fernández-Hidalgo N., Almirante B., Gavaldà J., Gurgui M., Peña C., de Alarcón A., Ruiz J., Vilacosta I., Montejo M., Vallejo N. (2013). Ampicillin Plus Ceftriaxone Is as Effective as Ampicillin Plus Gentamicin for Treating *Enterococcus faecalis* Infective Endocarditis. Clin. Infect. Dis..

[5] Gavaldà J., Len O., Miró J.M., Muñoz P., Montejo M., Alarcón A., de la Torre-Cisneros J., Peña C., Martínez-Lacasa X., Sarria C. (2007). Brief Communication: Treatment of Enterococcus Faecalis Endocarditis with Ampicillin plus Ceftriaxone. Ann. Intern. Med..

[6] Roberts J.A., Abdul-Aziz M.-H., Lipman J., Mouton J.W., Vinks A.A., Felton T.W., Hope W.W., Farkas A., Neely M.N., Schentag J.J. (2014). Individualised antibiotic dosing for patients who are critically ill: Challenges and potential solutions. Lancet Infect. Dis..

[7] Gatti M., Pea F. (2021). Continuous versus intermittent infusion of antibiotics in Gram-negative multidrug-resistant infections. Curr. Opin. Infect. Dis..

[8] Abdul-Aziz M.-H., Alffenaar J.-W.C., Bassetti M., Bracht H., Dimopoulos G., Marriott D., Neely M.N., Paiva J.-A., Pea F., Sjovall F. (2020). Antimicrobial therapeutic drug monitoring in critically ill adult patients: A Position Paper_#_. Intensive Care Med..

[9] Ogawa T., Kasahara K., Ikawa K., Shigeta J., Komatsu Y., Kuruno N., Uno K., Maeda K., Mikasa K. (2014). Continuous ampicillin infusion as an alternative to intermittent infusion for adult inpatients: A case series. J. Infect. Chemother..

[10] Lewis P.O., Jones A., Amodei R.J., Youssef D. (2020). Continuous Infusion Ampicillin for the Outpatient Management of Enterococcal Endocarditis: A Case Report and Literature Review. J. Pharm. Pract..

[11] Parsonson F., Legg M.A., Halford M.M., McCarthy K. (2020). Contemporaneous management of ampicillin infusions in the outpatient setting through the use of therapeutic drug monitoring. Int. J. Antimicrob. Agents.

[12] Li J.S., Sexton D.J., Mick N., Nettles R., Fowler V.G., Ryan T., Bashore T., Corey G.R. (2000). Proposed Modifications to the Duke Criteria for the Diagnosis of Infective Endocarditis. Clin. Infect. Dis..

[13] Gatti M., Cojutti P.G., Pascale R., Tonetti T., Laici C., Dell’Olio A., Siniscalchi A., Giannella M., Viale P., Pea F. (2021). Assessment of a PK/PD Target of Continuous Infusion Beta-Lactams Useful for Preventing Microbiological Failure and/or Resistance Development in Critically Ill Patients Affected by Documented Gram-Negative Infections. Antibiotics.

[14] Vardakas K.Z., Voulgaris G.L., Maliaros A., Samonis G., Falagas M.E. (2018). Prolonged versus short-term intravenous infusion of antipseudomonal β-lactams for patients with sepsis: A systematic review and meta-analysis of randomised trials. Lancet Infect. Dis..

[15] Thauvin C., Eliopoulos G.M., Willey S., Wennersten C., Moellering R.C. (1987). Continuous-infusion ampicillin therapy of enterococcal endocarditis in rats. Antimicrob. Agents Chemother..

[16] Ogawa T., Sato M., Yonekawa S., Nakagawa C., Uno K., Kasahara K., Maeda K., Konishi M., Mikasa K. (2013). Infective Endocarditis Caused by Enterococcus faecalis treated with Continuous Infusion of Ampicillin without Adjunctive Aminoglycosides. Intern. Med..

[17] Lupia T., Roberto G., Scaglione L., Shbaklo N., De Benedetto I., Scabini S., Pinna S.M., Curtoni A., Cavallo R., De Rosa F.G. (2022). Clinical and microbiological characteristics of bloodstream infections caused by *Enterococcus* spp. within internal medicine wards: A two-year single-centre experience. Intern. Emerg. Med..

[18] Crass R.L., Rodvold K.A., Mueller B.A., Pai M.P. (2019). Renal Dosing of Antibiotics: Are We Jumping the Gun?. Clin. Infect. Dis..

[19] Vardakas K.Z., Kalimeris G.D., Triarides N.A., Falagas M.E. (2018). An update on adverse drug reactions related to β-lactam antibiotics. Expert Opin. Drug Saf..

[20] Roger C., Louart B. (2021). Beta-Lactams Toxicity in the Intensive Care Unit: An Underestimated Collateral Damage?. Microorganisms.

[21] Pea F., Viale P. (2009). Bench-to-bedside review: Appropriate antibiotic therapy in severe sepsis and septic shock—Does the dose matter?. Crit. Care.

[22] Bussini L., Del Turco E.R., Pasquini Z., Scolz K., Amedeo A., Beci G., Giglia M., Tedeschi S., Pascale R., Ambretti S. (2022). Risk factors for persistent enterococcal bacteraemia: A multicentre retrospective study. J. Glob. Antimicrob. Resist..

[23] Sumi C.D., Heffernan A.J., Lipman J., Roberts J.A., Sime F.B. (2019). What Antibiotic Exposures Are Required to Suppress the Emergence of Resistance for Gram-Negative Bacteria? A Systematic Review. Clin. Pharmacokinet..

[24] Bartoletti M., Tedeschi S., Scudeller L., Pascale R., Rosselli Del Turco E., Trapani F., Tumietto F., Virgili G., Marconi L., Ianniruberto S. (2019). Impact on mortality of a bundle for the management of enterococcal bloodstream infection. Open Forum Infect. Dis..

[25] López-Cortés L.E., Del Toro M.D., Gálvez-Acebal J., Bereciartua-Bastarrica E., Fariñas M.C., Sanz-Franco M., Natera C., Corzo J.E., Lomas J.M., Pasquau J. (2013). Impact of an Evidence-Based Bundle Intervention in the Quality-of-Care Management and Outcome of Staphylococcus aureus Bacteremia. Clin. Infect. Dis..

[26] Paladino J.A., Poretz D. (2010). Outpatient Parenteral Antimicrobial Therapy Today. Clin. Infect. Dis..

[27] Barza M., Weinstein L. (1976). Pharmacokinetics of the Penicillins in Man. Clin. Pharmacokinet..

[28] Gatti M., Cojutti P.G., Bartoletti M., Tonetti T., Bianchini A., Ramirez S., Pizzilli G., Ambretti S., Giannella M., Mancini R. (2022). Expert clinical pharmacological advice may make an antimicrobial TDM program for emerging candidates more clinically useful in tailoring therapy of critically ill patients. Crit. Care.

